# Diaceto­nitrile­(2,2′-bi­pyridine-κ^2^ *N*,*N*′)palladium(II) bis­(tri­fluoro­methane­sulfonate)

**DOI:** 10.1107/S2414314622011518

**Published:** 2022-12-06

**Authors:** Rafael A. Adrian, Marcela C. Gutierrez, Hadi D. Arman

**Affiliations:** a Department of Chemistry and Biochemistry, University of the Incarnate Word, San Antonio, Texas 78209, USA; b Department of Chemistry, The University of Texas at San Antonio, San Antonio, Texas 78249, USA; Katholieke Universiteit Leuven, Belgium

**Keywords:** palladium, crystal structure, 2,2′-bi­pyridine, 2,2′-dipyrid­yl, bipy, coordinated aceto­nitrile, square planar, coordination complex

## Abstract

In the title complex, [Pd(C_10_H_8_N_2_)(CH_3_CN)_2_](CF_3_SO_3_)_2_ or [Pd(bipy)(CH_3_CN)_2_](CF_3_SO_3_)_2_, the palladium(II) ion is fourfold coordinated by two aceto­nitrile mol­ecules and a bidentate 2,2′-bi­pyridine ligand in a distorted square-planar geometry.

## Abstract 2

In the crystal structure of the title compound, the palladium(II) metal center is surrounded by a bidentate 2,2′-bi­pyridine ligand and two aceto­nitrile mol­ecules in a distorted square-planar geometry.

## Graphical abstract

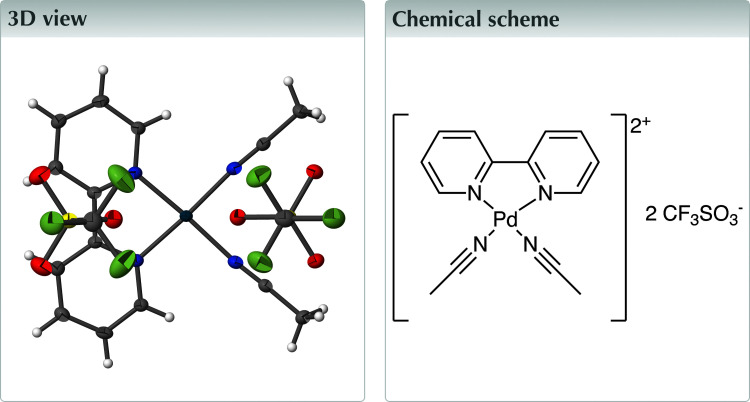

## Structure description

Palladium(II) complexes of 2,2′-bi­pyridine continue to be investigated due to their application in catalysis (Kitanosono *et al.*, 2021 ▸) and remarkable anti­proliferative activity against cancer cells (Fatahian-Nezhad *et al.*, 2021 ▸; Tabrizi *et al.*, 2020 ▸; Icsel *et al.*, 2015 ▸). Our research group has been exploring the synthesis of palladium(II) and copper(II) complexes containing various ancillary ligands. With that in mind, herein, we report the synthesis and structure of the title complex, an excellent starting material for synthesizing novel palladium complexes.

**Figure 1:**
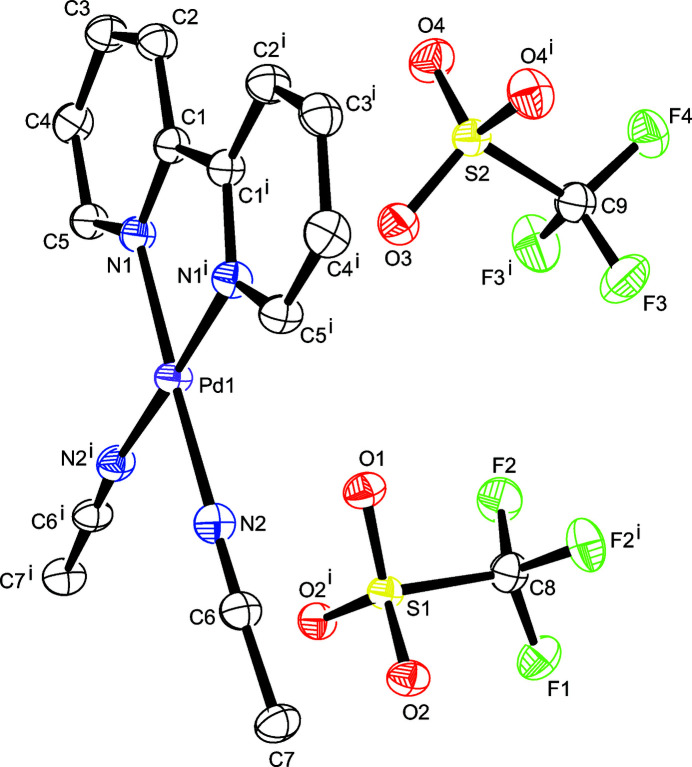
The mol­ecular structure of the title compound with displacement ellipsoids drawn at the 50% probability level; H atoms are omitted for clarity. Symmetry code: (i) *x*, −*y* + 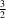 , *z*.

The asymmetric unit contains only half of the title complex due to the presence of a vertical plane of symmetry along the *a* axis that bis­ects the bond between the pyridine rings, C1—C1^i^, and the Pd1, O1, F1, O3, and F4 atoms. The title complex exhibits a Pd^II^ ion in a distorted square-planar coordination environment defined by two N atoms of the bidentate 2,2′-bi­pyridine ligand and one nitro­gen from each of the two coordinated aceto­nitrile mol­ecules. Two tri­fluoro­methane­sulfonate ions sit outside the coordination sphere of the title complex balancing the charge of the metal (Fig. 1 ▸). The Pd—N1 and Pd—N2 bond lengths of 1.999 (2) Å and 2.012 (3) Å, respectively, are in good agreement with the only comparable palladium(II) 2,2′-bi­pyridine complex, a tetra­fluoro­borate salt, (1.993 and 2.004 Å) currently available in the CSD (Groom *et al.*, 2016 ▸; version 5.43 with update of September 2022; refcode WEFCAL; Nesper *et al.*, 1993 ▸). The N—Pd—N angles also correlate well with the previously referenced complex, with differences of less than one degree in all cases. All relevant bonds and angles are presented in Table 1 ▸.

**Figure 2:**
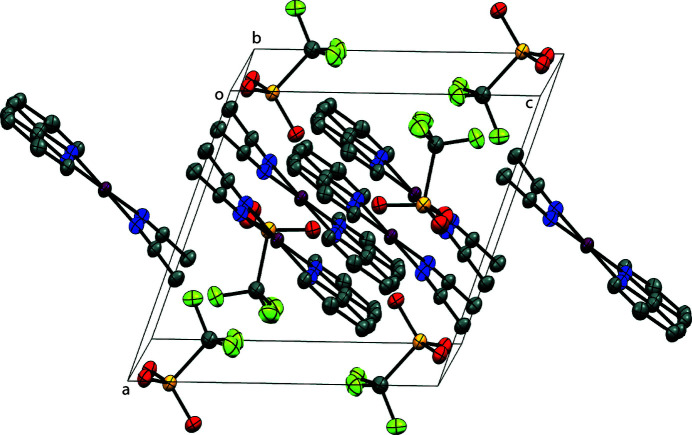
Perspective view of the packing structure of the title complex; H atoms are omitted for clarity.

In the molecular packing of the title complex, the palladium(II) complex ions align in layers along the *b*-axis orienting their coordinated aceto­nitrile toward the same direction, with the tri­fluoro­methane­sulfonate ions occupying the gaps between layers, as shown in Fig. 2 ▸. Meanwhile, adjacent layers alternate the orientation of the coordinated aceto­nitrile mol­ecules. No directional supra­mol­ecular inter­actions are present in the crystal packing of the title compound.

## Synthesis and crystallization

Silver tri­fluoro­methane­sulfonate (0.154 g, 0.600 mmol) was added to a 40.0 ml aceto­nitrile suspension of (2,2′-bi­pyridine)­dichloro­palladium(II) (0.100 g, 0.300 mmol). The resulting solution was filtrated using a 0.45 mm PTFE syringe filter and heated at 323 K to reduce the volume to 10.0 ml. Crystals suitable for X-ray diffraction were obtained by vapor diffusion of diethyl ether over the saturated aceto­nitrile solution of the title complex at 277 K.

## Refinement

Crystal data, data collection and structure refinement details are summarized in Table 2 ▸.

## Supplementary Material

Crystal structure: contains datablock(s) I. DOI: 10.1107/S2414314622011518/vm4055sup1.cif

Structure factors: contains datablock(s) I. DOI: 10.1107/S2414314622011518/vm4055Isup2.hkl

Supporting information file. DOI: 10.1107/S2414314622011518/vm4055Isup3.mol

CCDC reference: 2223394

Additional supporting information:  crystallographic information; 3D view; checkCIF report

## Figures and Tables

**Table 1:** Selected geometric parameters (Å, °)

Pd1—N1	1.999 (2)	Pd1—N2	2.012 (3)
			
N1^i^—Pd1—N1	81.82 (14)	N1—Pd1—N2	176.99 (9)
N1—Pd1—N2^i^	95.18 (11)	N2—Pd1—N2^i^	87.83 (14)

Symmetry code: (i) 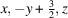 .

**Table 2:** Experimental details

Crystal data	
Chemical formula	[Pd(C_10_H_8_N_2_)(C_2_H_3_N)_2_](CF_3_O_3_S)_2_
*M*_r_	642.83
Crystal system, space group	Monoclinic, *P*2_1_/*m*
Temperature (K)	100
*a*, *b*, *c* (Å)	9.2732 (3), 12.5307 (2), 10.0983 (3)
β (°)	110.627 (3)
*V* (Å^3^)	1098.20 (6)
*Z*	2
Radiation type	Cu *K*α
μ (mm^−1^)	9.49
Crystal size (mm)	0.15 × 0.13 × 0.09

Data collection	
Diffractometer	XtaLAB Synergy, Dualflex, HyPix
Absorption correction	Gaussian (*CrysAlis PRO*; Rigaku OD, 2020 ▸)
*T*_min_, *T*_max_	0.746, 1.000
No. of measured, independent and observed [*I* > 2σ(*I*)] reflections	10720, 2318, 2234
*R*_int_	0.048
(sin θ/λ)_max_ (Å^−1^)	0.630

Refinement	
*R*[*F* ^2^ > 2σ(*F* ^2^)], *wR*(*F* ^2^), *S*	0.033, 0.093, 1.09
No. of reflections	2318
No. of parameters	174
H-atom treatment	H-atom parameters constrained
Δρ_max_, Δρ_min_ (e Å^−3^)	0.75, −0.93

Computer programs: *CrysAlis PRO* (Rigaku OD, 2020 ▸), *SHELXT2018/2* (Sheldrick, 2015*a* ▸), *SHELXL2018/3* (Sheldrick, 2015*b* ▸), and *OLEX2* (Dolomanov *et al.*, 2009 ▸).

## full crystallographic data

### Crystal data

**Table:**

[Pd(C_10_H_8_N_2_)(C_2_H_3_N)_2_](CF_3_O_3_S)_2_	*F*(000) = 636
*M**_r_* = 642.83	*D*_x_ = 1.944 Mg m^−^^3^
Monoclinic, *P*12_1_/*m*1	Cu *K*α radiation, λ = 1.54184 Å
*a* = 9.2732 (3) Å	Cell parameters from 5466 reflections
*b* = 12.5307 (2) Å	θ = 4.7–75.7°
*c* = 10.0983 (3) Å	µ = 9.49 mm^−^^1^
β = 110.627 (3)°	*T* = 100 K
*V* = 1098.20 (6) Å^3^	Block, clear colourless
*Z* = 2	0.15 × 0.13 × 0.09 mm

### Data collection

**Table:**

XtaLAB Synergy, Dualflex, HyPix diffractometer	2318 independent reflections
Radiation source: micro-focus sealed X-ray tube, PhotonJet (Cu) X-ray Source	2234 reflections with *I* > 2σ(*I*)
Mirror monochromator	*R*_int_ = 0.048
Detector resolution: 10.0000 pixels mm^-1^	θ_max_ = 76.3°, θ_min_ = 4.7°
ω scans	*h* = −11→11
Absorption correction: gaussian (CrysAlisPro; Rigaku OD, 2020)	*k* = −11→15
*T*_min_ = 0.746, *T*_max_ = 1.000	*l* = −12→12
10720 measured reflections	

### Refinement

**Table:**

Refinement on *F*^2^	Hydrogen site location: inferred from neighbouring sites
Least-squares matrix: full	H-atom parameters constrained
*R*[*F*^2^ > 2σ(*F*^2^)] = 0.033	*w* = 1/[σ^2^(*F*_o_^2^) + (0.0618*P*)^2^ + 0.5328*P*] where *P* = (*F*_o_^2^ + 2*F*_c_^2^)/3
*wR*(*F*^2^) = 0.093	(Δ/σ)_max_ < 0.001
*S* = 1.09	Δρ_max_ = 0.75 e Å^−^^3^
2318 reflections	Δρ_min_ = −0.93 e Å^−^^3^
174 parameters	Extinction correction: *SHELXL2018/3* (Sheldrick, 2015*b*), Fc^*^=kFc[1+0.001xFc^2^λ^3^/sin(2θ)]^-1/4^
0 restraints	Extinction coefficient: 0.00084 (18)
Primary atom site location: dual	

### Special details

**Table:**

Geometry. All esds (except the esd in the dihedral angle between two l.s. planes) are estimated using the full covariance matrix. The cell esds are taken into account individually in the estimation of esds in distances, angles and torsion angles; correlations between esds in cell parameters are only used when they are defined by crystal symmetry. An approximate (isotropic) treatment of cell esds is used for estimating esds involving l.s. planes.

### Fractional atomic coordinates and isotropic or equivalent isotropic displacement parameters (Å^2^)

**Table:**

	*x*	*y*	*z*	*U*_iso_*/*U*_eq_	
Pd1	0.45413 (3)	0.750000	0.68769 (3)	0.01791 (13)	
S1	0.95842 (11)	0.750000	0.87020 (10)	0.0194 (2)	
S2	0.58391 (11)	0.750000	0.25835 (10)	0.0222 (2)	
F1	1.2500 (3)	0.750000	0.8920 (3)	0.0318 (6)	
F2	1.0991 (2)	0.66371 (16)	0.7117 (2)	0.0362 (5)	
F4	0.8145 (3)	0.750000	0.1642 (3)	0.0343 (6)	
F3	0.8599 (2)	0.83614 (19)	0.3585 (2)	0.0455 (5)	
O2	0.9882 (2)	0.84692 (17)	0.9529 (2)	0.0240 (4)	
O1	0.8188 (3)	0.750000	0.7478 (3)	0.0250 (6)	
O3	0.5802 (4)	0.750000	0.3998 (3)	0.0276 (6)	
O4	0.5311 (3)	0.65247 (18)	0.1802 (2)	0.0332 (5)	
N1	0.3443 (3)	0.64555 (19)	0.5361 (2)	0.0201 (5)	
N2	0.5598 (3)	0.8614 (2)	0.8330 (3)	0.0220 (5)	
C1	0.2630 (3)	0.6910 (2)	0.4102 (3)	0.0192 (5)	
C5	0.3494 (3)	0.5388 (2)	0.5486 (3)	0.0218 (6)	
H5	0.405311	0.508152	0.635496	0.026*	
C4	0.2733 (4)	0.4734 (2)	0.4351 (3)	0.0254 (6)	
H4	0.277737	0.399608	0.445345	0.030*	
C6	0.6305 (3)	0.9177 (2)	0.9187 (3)	0.0219 (6)	
C2	0.1852 (3)	0.6298 (2)	0.2937 (3)	0.0241 (6)	
H2	0.129953	0.662005	0.207740	0.029*	
C3	0.1905 (3)	0.5194 (2)	0.3063 (3)	0.0248 (6)	
H3	0.138764	0.476742	0.228681	0.030*	
C7	0.7208 (4)	0.9927 (2)	1.0251 (3)	0.0255 (6)	
H7A	0.688876	1.064212	0.994367	0.038*	
H7B	0.827941	0.984372	1.038591	0.038*	
H7C	0.705270	0.979190	1.112678	0.038*	
C9	0.7904 (5)	0.750000	0.2872 (5)	0.0285 (9)	
C8	1.1090 (5)	0.750000	0.7920 (5)	0.0265 (9)	

### Atomic displacement parameters (Å^2^)

**Table:**

	*U*^11^	*U*^22^	*U*^33^	*U*^12^	*U*^13^	*U*^23^
Pd1	0.02209 (19)	0.01259 (19)	0.01685 (19)	0.000	0.00414 (13)	0.000
S1	0.0234 (5)	0.0146 (5)	0.0189 (4)	0.000	0.0057 (4)	0.000
S2	0.0243 (5)	0.0181 (5)	0.0229 (5)	0.000	0.0067 (4)	0.000
F1	0.0236 (13)	0.0283 (14)	0.0400 (14)	0.000	0.0067 (11)	0.000
F2	0.0426 (11)	0.0326 (10)	0.0392 (11)	−0.0031 (9)	0.0217 (9)	−0.0121 (8)
F4	0.0387 (15)	0.0366 (15)	0.0341 (14)	0.000	0.0210 (12)	0.000
F3	0.0350 (11)	0.0549 (13)	0.0496 (13)	−0.0183 (10)	0.0187 (10)	−0.0220 (11)
O2	0.0303 (11)	0.0174 (10)	0.0225 (10)	0.0003 (8)	0.0069 (8)	−0.0030 (8)
O1	0.0236 (14)	0.0221 (15)	0.0248 (14)	0.000	0.0030 (11)	0.000
O3	0.0294 (16)	0.0280 (16)	0.0267 (15)	0.000	0.0113 (13)	0.000
O4	0.0362 (12)	0.0276 (12)	0.0357 (12)	−0.0069 (10)	0.0127 (10)	−0.0090 (9)
N1	0.0230 (11)	0.0161 (11)	0.0202 (11)	−0.0015 (9)	0.0065 (9)	0.0007 (9)
N2	0.0247 (12)	0.0168 (12)	0.0235 (12)	0.0027 (10)	0.0070 (10)	0.0021 (10)
C1	0.0212 (13)	0.0152 (14)	0.0207 (13)	−0.0006 (11)	0.0070 (11)	−0.0001 (10)
C5	0.0257 (14)	0.0171 (14)	0.0209 (13)	−0.0009 (11)	0.0061 (11)	0.0011 (11)
C4	0.0312 (15)	0.0154 (13)	0.0294 (14)	−0.0029 (12)	0.0104 (12)	−0.0021 (11)
C6	0.0251 (14)	0.0174 (14)	0.0205 (13)	0.0012 (11)	0.0045 (11)	0.0029 (11)
C2	0.0263 (14)	0.0219 (15)	0.0221 (13)	−0.0006 (12)	0.0060 (11)	0.0007 (11)
C3	0.0279 (14)	0.0214 (15)	0.0240 (13)	−0.0018 (12)	0.0078 (11)	−0.0048 (11)
C7	0.0281 (15)	0.0195 (14)	0.0249 (14)	−0.0041 (12)	0.0043 (12)	−0.0025 (12)
C9	0.028 (2)	0.029 (2)	0.027 (2)	0.000	0.0092 (18)	0.000
C8	0.028 (2)	0.022 (2)	0.029 (2)	0.000	0.0104 (18)	0.000

### Geometric parameters (Å, º)

**Table:**

Pd1—N1^i^	1.999 (2)	N1—C1	1.354 (4)
Pd1—N1	1.999 (2)	N1—C5	1.343 (4)
Pd1—N2	2.012 (3)	N2—C6	1.130 (4)
Pd1—N2^i^	2.012 (3)	C1—C1^i^	1.480 (5)
S1—O2^i^	1.444 (2)	C1—C2	1.376 (4)
S1—O2	1.444 (2)	C5—H5	0.9300
S1—O1	1.441 (3)	C5—C4	1.383 (4)
S1—C8	1.830 (4)	C4—H4	0.9300
S2—O3	1.441 (3)	C4—C3	1.382 (4)
S2—O4^i^	1.443 (2)	C6—C7	1.451 (4)
S2—O4	1.444 (2)	C2—H2	0.9300
S2—C9	1.833 (5)	C2—C3	1.389 (4)
F1—C8	1.341 (5)	C3—H3	0.9300
F2—C8	1.335 (3)	C7—H7A	0.9600
F4—C9	1.338 (5)	C7—H7B	0.9600
F3—C9	1.331 (3)	C7—H7C	0.9600
			
N1^i^—Pd1—N1	81.82 (14)	C5—C4—H4	120.5
N1^i^—Pd1—N2	95.18 (11)	C3—C4—C5	119.0 (3)
N1—Pd1—N2^i^	95.18 (11)	C3—C4—H4	120.5
N1—Pd1—N2	176.99 (9)	N2—C6—C7	178.1 (3)
N1^i^—Pd1—N2^i^	176.99 (9)	C1—C2—H2	120.5
N2—Pd1—N2^i^	87.83 (14)	C1—C2—C3	119.0 (3)
O2—S1—O2^i^	114.47 (17)	C3—C2—H2	120.5
O2^i^—S1—C8	103.28 (12)	C4—C3—C2	119.4 (3)
O2—S1—C8	103.28 (12)	C4—C3—H3	120.3
O1—S1—O2	115.28 (10)	C2—C3—H3	120.3
O1—S1—O2^i^	115.28 (11)	C6—C7—H7A	109.5
O1—S1—C8	102.79 (19)	C6—C7—H7B	109.5
O3—S2—O4^i^	114.85 (11)	C6—C7—H7C	109.5
O3—S2—O4	114.85 (11)	H7A—C7—H7B	109.5
O3—S2—C9	103.35 (19)	H7A—C7—H7C	109.5
O4^i^—S2—O4	115.7 (2)	H7B—C7—H7C	109.5
O4^i^—S2—C9	102.76 (12)	F4—C9—S2	111.1 (3)
O4—S2—C9	102.75 (12)	F3—C9—S2	111.4 (2)
C1—N1—Pd1	114.14 (18)	F3^i^—C9—S2	111.4 (2)
C5—N1—Pd1	126.1 (2)	F3—C9—F4	107.2 (2)
C5—N1—C1	119.8 (2)	F3^i^—C9—F4	107.2 (2)
C6—N2—Pd1	173.7 (2)	F3—C9—F3^i^	108.4 (4)
N1—C1—C1^i^	114.85 (15)	F1—C8—S1	111.4 (3)
N1—C1—C2	121.3 (2)	F2^i^—C8—S1	111.2 (2)
C2—C1—C1^i^	123.82 (17)	F2—C8—S1	111.2 (2)
N1—C5—H5	119.3	F2—C8—F1	107.3 (2)
N1—C5—C4	121.4 (3)	F2^i^—C8—F1	107.3 (2)
C4—C5—H5	119.3	F2^i^—C8—F2	108.2 (3)
			
Pd1—N1—C1—C1^i^	−3.37 (19)	O4—S2—C9—F4	60.23 (11)
Pd1—N1—C1—C2	177.7 (2)	O4^i^—S2—C9—F4	−60.23 (11)
Pd1—N1—C5—C4	−177.6 (2)	O4—S2—C9—F3^i^	−59.2 (3)
O2^i^—S1—C8—F1	−59.77 (10)	O4^i^—S2—C9—F3	59.2 (3)
O2—S1—C8—F1	59.77 (10)	O4^i^—S2—C9—F3^i^	−179.6 (2)
O2^i^—S1—C8—F2^i^	−179.4 (2)	O4—S2—C9—F3	179.6 (2)
O2—S1—C8—F2^i^	−59.9 (3)	N1—C1—C2—C3	0.1 (4)
O2—S1—C8—F2	179.4 (2)	N1—C5—C4—C3	0.1 (4)
O2^i^—S1—C8—F2	59.9 (3)	C1—N1—C5—C4	0.1 (4)
O1—S1—C8—F1	180.000 (1)	C1^i^—C1—C2—C3	−178.68 (19)
O1—S1—C8—F2	−60.3 (2)	C1—C2—C3—C4	0.1 (4)
O1—S1—C8—F2^i^	60.3 (2)	C5—N1—C1—C1^i^	178.68 (19)
O3—S2—C9—F4	180.000 (1)	C5—N1—C1—C2	−0.2 (4)
O3—S2—C9—F3	−60.6 (2)	C5—C4—C3—C2	−0.2 (4)
O3—S2—C9—F3^i^	60.6 (2)		

Symmetry code: (i) *x*, −*y*+3/2, *z*.
